# From Rosalind Franklin to Barack Obama: Data Sharing Challenges and Solutions in Genomics and Personalised Medicine[Fn FN0000]

**DOI:** 10.1080/20502877.2017.1314883

**Published:** 2017-05-18

**Authors:** Mark Lawler, Tim Maughan

**Affiliations:** ^a^ Centre for Cancer Research, Queen's University Belfast, Belfast, UK; ^b^ Clinical Working Group, Global Alliance for Genomics and Health, Boston, USA; ^c^ CRUK/MRC Oxford Institute for Radiation Oncology, University of Oxford, Oxford, UK

**Keywords:** personalised medicine, big data, data sharing, genomics, cancer, bioethics

## Abstract

The collection, storage and use of genomic and clinical data from patients and healthy individuals is a key component of personalised medicine enterprises such as the Precision Medicine Initiative, the Cancer Moonshot and the 100,000 Genomes Project. In order to maximise the value of this data, it is important to embed a culture within the scientific, medical and patient communities that supports the appropriate sharing of genomic and clinical information. However, this aspiration raises a number of ethical, legal and regulatory challenges that need to be addressed. The Global Alliance for Genomics and Health, a worldwide coalition of researchers, healthcare professionals, patients and industry partners, is developing innovative solutions to support the responsible and effective sharing of genomic and clinical data. This article identifies the challenges that a data sharing culture poses and highlights a series of practical solutions that will benefit patients, researchers and society.

## A cautionary tale: bioethics and the discovery of ‘the secret of life’

Sharing data can lead to new knowledge, but what about consent?

In the Eagle Pub in Cambridge in England, a metal plaque on the wall bears the following inscription:

On this spot, on February 28^th^ 1953, Frances Crick and James Watson made the first public announcement of the discovery of DNA, with the words ‘We have discovered the secret of life’.

This of course led to their landmark paper in Nature (Watson and Crick 1953) in 1953 and the subsequent award of the Nobel Prize for Physiology or Medicine to Crick, Watson and their colleague Maurice Wilkins in 1962.

A second plaque, slightly lower down on the same wall, has the following epitaph:

In memory of Rosalind Franklin (Newnham College 1938–1941) whose exceptionally skilled crystallography enabled Crick and Watson to unravel the double helix structure of DNA. Tragically she died before the Nobel Prize was awarded. Her contribution was not fully recognised until much later

Franklin's key contribution was ‘Photograph 51’, the nickname given to the X-ray diffraction image taken by her and her PhD student Raymond Gosling in May 1952. This iconic image was subsequently shown by Maurice Wilkins (without Franklin's knowledge or more importantly her consent) to Watson and Crick, who recognised its significance and began building potential models that captured the double helical structure of Deoxyribonucleic acid (DNA), leading to their prophetic announcement in the Eagle Pub some 9 months later.

## Secondary use of data: parasitism or symbiosis

Viewing this somewhat controversial aspect of the discovery of DNA through a bioethics prism highlights the challenges that genetic/genomic research has faced and continues to experience in the context of data generation and its (myriad) uses. Spinning the helix forward to 2016, the rather unfortunate characterisation of secondary users of data as ‘research parasites’ by Dan Longo and Jeffrey Drazen (deputy editor and editor of the New England Journal of Medicine respectively) (Longo and Drazen 2016a) led to a series of letters to the editors of a number of medical and scientific journals (including the New England Journal of Medicine) and a series of blogs on the issue, prompting a more nuanced reassessment by Longo and Drazen of the relationship between primary data producers and data (re)analysts (Longo and Drazen 2016b). A number of key questions include — Who owns the data? How can it be used? How do we preserve the rights of the original data source/ data generator? All are highly pertinent to medical research and its clinical translation and need to be addressed.

## Data sharing at scale: big science, big data and big challenges

Data mining and the potential for data sharing form a key component of a number of recent high profile genomics/personalised medicine initiatives that also have significant clinical potential. In his State of the Union address in 2015, former US President Barack Obama announced his intention to launch the Precision Medicine Initiative (PMI) (The White House, https://www.whitehouse.gov/the-press-office/2015/01/30/fact-sheet-president-obama-s-precision-medicine-initiative), an innovative programme which aims to move away from the ‘one size fits all’ approach for therapeutic intervention and employ a more precise knowledge of the genomic, clinical and epidemiological characteristics of the patient's individual disease to inform treatment decision-making. As part of the initiative and with specific reference to cancer, he highlighted the need for the development of a ‘cancer knowledge network’ as the ideal mechanism to best manage and utilise the huge amount of data that will be generated through PMI.

Cancer was also very much the focus of then US Vice President Joe Biden when he was addressing the American Society for Clinical Oncology Conference in Chicago last June. Speaking of his vision for the Cancer Moonshot (ASCO, http://am.asco.org/vice-president-joe-biden-discusses-cancer-moonshot-initiative-during-asco-2016) an initiative to accelerate cancer research, such that this killer disease can be either prevented, detected earlier or treated more successfully, he announced the launch of the National Cancer Institute's Genomic Data Commons (NCI-GDC) (NIH, https://www.nih.gov/news-events/news-releases/newly-launched-genomic-data-commons-facilitate-data-clinical-information-sharing; Grossman *et al.* 2016), a valuable research resource that contains significant quantities of data from NCI sponsored research, with over four petabytes of data initially being released to the global cancer community to allow comprehensive mining of this information.

Two recent research publications with clear clinical relevance have reported on the linkage of genomic data from 50,000 patients with their Electronic Health Records (EHRs). This work forms part of the DiscoverEHR study of the Geisinger MyCode Community Health initiative and highlight the ability to combine DNA sequencing data with clinical information to identify novel therapeutic targets, in this case particularly in cardiovascular diseases (Abul-Husn *et al.* 2016; Dewey *et al.* 2016).

In the UK, the 100,000 Genomes Project, an initiative that was introduced by ex-Prime Minister David Cameron, is generating significant amounts of genomic and clinical data that will inform disease diagnosis and treatment decision-making, particularly for rare diseases and cancer (Siva 2015). There are similar national efforts at different stages of development across the world.

However, for all of these initiatives, there are many challenges, including significant ethical and legal issues that need to be addressed, in order to ensure that data is shared and utilised in an appropriate fashion.

## Ensuring responsible and effective sharing of data: the Global Alliance for Genomics and Health

So how do we ensure, at an international level, that data sharing which is beneficial to the individual and to society can occur, in a way in which the rights of the individual (and/or the data producer) are appropriately addressed and the legal and ethical responsibilities are clearly delineated? The Global Alliance for Genomics and Health (GA4GH) (Global Alliance for Genomics and Health, https://genomicsandhealth.org/about-the-global-alliance/key-documents/white-paper-creating-global-alliance-enable-responsible-shar; Global Alliance for Genomics and Health *et al.* 2016) is an international coalition of life and data scientists, clinicians, patients and their advocates and information technology/bio industry partners, dedicated to promoting responsible and effective sharing of genomic and clinical data. Since its inaugural conference in the Welcome Trust in London in 2013, GA4GH has grown significantly and now has over 450 institutional members from >60 countries (Global Alliance for Genomics and Health, https://genomicsandhealth.org/members), with a clear focus on genomic and clinical data sharing in rare disease and in cancer, and an emerging interest in infectious disease. The activities of GA4GH are mediated through four Working Groups: The Clinical Working Group, the Data Working Group, the Regulatory and Ethics Working Group and the Security Working Group. In addition, a series of Task Teams, dedicated to particular issues or challenges, develop and deliver particular projects, scientific and policy papers and tools/solutions that promote and simplify the sharing of data.

## Changing the mindset: from selfish silo to collaborative culture

One of the challenges that can undermine effective linking of multiple sources of information is when the data repositories that would benefit from being interlinked and appropriately mined to deliver added value, instead sit in siloed isolation with no ability to ‘talk’ to each other (Lawler *et al.* 2015). Establishing a flourishing data sharing culture in diseases such as cancer also requires a collective change in the individual researcher mindset, moving away from this closed ‘selfish silo' approach to embrace a more ‘open source' collaborative culture. Reluctance to share data in academia is, in some cases, influenced by perceptions of loss of control and dilution of credit in multi-author studies. The pressures of increased competition for research funding and the need for senior authored research papers to satisfy institutional academic standards, allied to the contribution of these two metrics to support applications for academic promotion, can negatively affect the appetite of researchers to pool data for mutual benefit. However, working together in data sharing projects enhances scientific rigour, increases the statistical power of the particular study and delivers rich sources of reliable data. Ensuring credit for collaborative data sharing efforts can be facilitated using appropriate microattribution-based credits (Patrinos *et al.* 2012), (akin to a ‘Genomic Bitcoin’) such that data producers and data consumers both receive fair reward for their research contribution and effort. An even greater resistance to data sharing has pervaded bio-industry, leading to unnecessary duplication of effort, which in a number of cases, has yielded negative results (Turner *et al.* 2008), wasting millions of dollars and precious patient resources, while delivering little discernable clinical benefit. In this regard, the Innovative Medicines Initiative (IMI), the public private partnership between the European Commission and Industry is driving an initiative that is breaking down some of these data silos. Through its *Big Data for Better Health* funding programme (Innovative Medicines Initiatives, http://www.imi.europa.eu/content/imi-2-call-6-launch), industry and academia are working together to share data in order to maximise its value in enhancing human health. The Structural Genomics Consortium (http://www.thesgc.org/) represents another public private partnership, between six academic institutions and industry, promoting an open access research culture that shares the fruits of its labour in developing new medicines through an ‘Innovation for All' philosophy.

## Ensuring ethical rigour: the framework for responsible sharing of genomic and health related data

Data sharing, particularly across different countries/jurisdictions may be challenging within a traditional ethics framework, particularly given the global diversity in legal and regulatory requirements. Additionally, potential privacy issues may be more prevalent, given the ability of genetic material to identify an individual person or a member of their family. To ensure that data sharing efforts and cross border open source collaborative approaches are developed and applied with appropriate ethical rigour, GA4GH has considered ethical issues from a fundamental human rights perspective, by developing a *Framework for Responsible Sharing of Genomic and Health Related Data* (Global Alliance for Genomics and Health, https://genomicsandhealth.org/about-the-global-alliance/key-documents/framework-responsible-sharing-genomic-and-health-related-data). This perspective extends the traditional bioethics approach within a harmonised framework that preserves the rights of the individual, but addresses current ‘No Can Do' defensive data access practices by firmly espousing the ‘Yes We Can' right of the individual (patient or researcher) to benefit from the increased outputs of research, mediated through appropriate and secure data sharing solutions. Employing a legal human rights perspective can circumvent many of the challenges that a traditional ethics framework may present, positioning genomic and clinical data sharing within an international legal framework that provides support for data sharing for the benefit of patients, thus encouraging adherence to the Framework principles by all stakeholders while offering legal protection to patients in areas such as fair access, privacy and discrimination (Knoppers 2014).

Underpinning this Framework are a series of overarching ethics policies. These include the GA4GH Consent Policy (Global Alliance for Genomics and Health, https://genomicsandhealth.org/consent-policy-pdf-27-may-2015), which provides useful guidance on the principles and practice of ensuring appropriate consent in different data sharing scenarios. In addition, GA4GH has created something akin to a Data Sharing Ethics ‘Toolbox’, which includes the following consent tools (i) *A Legacy Consent and International Data Sharing Tool* (GA P3G-IPAC Consent Tools, https://genomicsandhealth.org/ga-p3g-ipac-consent-tools) which addresses the issue of consents that were taken at the time of the original study, but which may not have considered/envisaged the diversity and complexity of future scientific use(s) of samples or data); (ii) *A Generic International Data Sharing Prospective Consent Form* (GA P3G-IPAC Consent Tools, https://genomicsandhealth.org/ga-p3g-ipac-consent-tools) which provides a readily adaptable consent template for international data sharing approaches for prospective studies; and (iii) A series of *Clauses for International Data Sharing* (GA P3G-IPAC Consent Tools, https://genomicsandhealth.org/ga-p3g-ipac-consent-tools) which contribute both useful advice and a series of templates for researchers who wish to add appropriate clauses or addendums in relation to international data sharing to their existing consent document(s). Portable and/or electronic consent tools are also under development. These policies and tools help deliver appropriate support, advice and relevant templates for researchers to ensure that their data sharing activities are conducted with due respect to rigorous ethical and legal principles.

GA4GH's Regulatory and Ethics Working Group has produced both a *Privacy and Security Policy* (Global Alliance for Genomics and Health, https://genomicsandhealth.org/privacy-and-security-policy-pdf-26-may-2015) and a ‘safe harbor' mechanism for privacy protection (Knoppers *et al.* 2014), which can be tailored to address particular scenarios such as those in relation to cross-border data sharing (Siu *et al.* 2016). In addition, the GA4GH Security Working Group has created a *Security Infrastructure Policy Paper* (Global Alliance for Genomics and Health, https://genomicsandhealth.org/security-infrastructure-version-11) which specifically address privacy and security concerns, delineating the appropriate standards and implementation practices that are required in order to protect the privacy and security of shared genomic and clinical data.

## Data repository and data analysis solutions: centralised versus federated approaches

A second relevant area that has been considered by the GA4GH data sharing community is the requirement for a trusted data sharing environment, which allows linking of databases in different countries/jurisdictions through a common network. This is especially relevant given the increasingly complex legal environment that can limit transfer of data across borders. Rather than moving the data between countries/jurisdictions, GA4GH's guiding principle is that the data repositories stay in the location where they have been generated (i.e. a federated rather than a centralised approach) and appropriate analysis tools are ‘ported’ to the data and perform their analysis ‘on site’. In this way, data privacy and security of the primary dataset are ensured, thus satisfying any ethical/legal concerns of the data hosting institution or consortium, while the data consumer is able to perform appropriate analysis on the rich information source. At a technical level, GA4GH has also developed a series of analysis tools including the GA4GH Application Programing Interface (API) (Global Alliance for Genomics and Health, https://genomicsandhealth.org/work-products-demonstration-projects/genomics-api), which provides common web based protocols to help in data sharing and analysis efforts.

## Free the data — cancer patients want their data to be shared

Critical to the establishment of a vibrant data sharing culture is the cancer patient. As highlighted in the European Cancer Patient's Bill of Rights (Lawler *et al.* 2014) when it was launched in the European Parliament in Strasbourg on World Cancer Day 2014 (European Cancer Concord, http://www.europeancancerconcord.eu/), it is becoming clear that cancer patients are no longer passive recipients in both research and its clinical translation but are increasingly becoming active participants. Despite the potential challenges that the recently approved General Data Protection Regulation presents to performing research involving patients (and indeed healthy individuals) in Europe (European Commission, http://ec.europa.eu/justice/data-protection/document/review2012/com_2012_11_en.pdf), there is increasing evidence that cancer patients want their data to be shared (www.free-the-data.org/) (Lin *et al.* 2004; Rogith *et al.* 2014). In addition, a number of cancer patient communities are now self-aggregating online to share their genomic data, in the hope of developing patient-led clinical trials, particularly for rarer mutations that may be targetable by a personalised medicine approach (GRACE, http://cancergrace.org/lung/2016/07/08/asco-2016-can-online-patient-groups-speed-the-development-of-new-targeted-therapies/). As patients increasingly provide their tissues and their data to enhance scientific research from which they individually, in the majority of cases, will not benefit, there is now a moral imperative to ensure that the research community follows suit, and desists from hoarding data in scientific silos, in favour of an open access collaborative approach that maximises the value of the research data generated. Academic research is predominantly supported through public funding, prompting funding agencies to increasingly require that the data generated is made available through open access as quickly as possible to the scientific community.

In conclusion, sharing data in a common disease such as cancer raises complex ethical issues that need to be addressed in a sensitive and appropriate fashion. However, by involving all stakeholders in what might be termed a ‘coalition of the willing’ and ensuring that appropriate ethical frameworks are developed and rigorously applied, we can provide a conduit for the responsible and effective sharing of genomic and clinical data for the benefit of patients, researchers and society.

## Related video

A video linked to this paper can be found at: http://www.healthcarevalues.ox.ac.uk/mark-lawler-data-sharing-and-participation
